# Single Plane Illumination Microscopy for Microfluidic Device Imaging

**DOI:** 10.3390/bios12121110

**Published:** 2022-12-01

**Authors:** Clara Gomez-Cruz, Sonia Laguna, Ariadna Bachiller-Pulido, Cristina Quilez, Marina Cañadas-Ortega, Ignacio Albert-Smet, Jorge Ripoll, Arrate Muñoz-Barrutia

**Affiliations:** 1 Departamento de Bioingeniería e Ingeniería Aeroespacial, Universidad Carlos III de Madrid, 28911 Leganés, Spain; 2 Instituto de Investigación Sanitaria Gregorio Marañón, 28009 Madrid, Spain

**Keywords:** light sheet fluorescence microscopy, microfluidic devices, live-cell imaging

## Abstract

Three-dimensional imaging of live processes at a cellular level is a challenging task. It requires high-speed acquisition capabilities, low phototoxicity, and low mechanical disturbances. Three-dimensional imaging in microfluidic devices poses additional challenges as a deep penetration of the light source is required, along with a stationary setting, so the flows are not perturbed. Different types of fluorescence microscopy techniques have been used to address these limitations; particularly, confocal microscopy and light sheet fluorescence microscopy (LSFM). This manuscript proposes a novel architecture of a type of LSFM, single-plane illumination microscopy (SPIM). This custom-made microscope includes two mirror galvanometers to scan the sample vertically and reduce shadowing artifacts while avoiding unnecessary movement. In addition, two electro-tunable lenses fine-tune the focus position and reduce the scattering caused by the microfluidic devices. The microscope has been fully set up and characterized, achieving a resolution of 1.50 μm in the x-y plane and 7.93 μm in the z-direction. The proposed architecture has risen to the challenges posed when imaging microfluidic devices and live processes, as it can successfully acquire 3D volumetric images together with time-lapse recordings, and it is thus a suitable microscopic technique for live tracking miniaturized tissue and disease models.

## 1. Introduction

Microfluidics refers to the technologies that allow the manipulation of small fluid volumes using fabricated microsystems [1]. Microfluidic devices provide several advantageous features for biomedical applications. The first approaches were mainly used as analytical tools, providing faster and cheaper results than conventional techniques. Namely, processes are more affordable as they require less sample and reagent volume. Another common use of microfluidic devices is the development of flow cytometers, used for counting different types of particles or cells [2,3]. More recently, organ-on-a-chip microfluidic devices have been developed to model human tissues in vitro. Organ-on-a-chip devices aim to reproduce the functionality or structure of an organ using microchannels, extracellular matrix and the pertinent cells [4].

Although many different materials are used to fabricate microdevices, polydimethylsiloxane (PDMS) continues to be the most used in microfluidics biomedical research [5,6]. The main advantage of using PDMS is the ease of fabrication through soft lithography techniques, which allows for fast prototyping. Regarding biomedical applications for microfluidics, PDMS is biocompatible, permeable to gas exchange and transparent, allowing sample imaging throughout the experiments [7,8].

A big challenge in using microfluidics for biomedical research, particularly for cell culture, is imaging inside the microdevices. Specifically, the need for light to transverse the PDMS forming the walls of the channels and the hydrogels or other compounds in which cells might be embedded. The main requirement for microscopy techniques to work in microdevices is an ability to penetrate deep and highly scattering samples and provide good optical sectioning capabilities for 3D cell cultures. Other requirements include the acquisition of large fields of view, fast imaging, and low phototoxicity to reduce photobleaching during live imaging. Epifluorescence microscopy can be a valuable tool for providing a general overview of the samples. However, it fails to provide information on the vertical location of the signal. Classical laser scanning confocal microscopy provides optical sectioning at the expense of slow acquisition and high phototoxicity [9]. An advantage of confocal microscopy for imaging microfluidic devices is that they are usually mounted on standard microscopy slides, so there is no PDMS in the light path, reducing the light scattering. However, its main disadvantage is its inability to penetrate more than a few cell layers, which means that some regions of the samples cannot be adequately illuminated [10,11].

Novel microscopes have been proposed that aim to overcome the disadvantages discussed above. For instance, computational clearing has been used to eliminate out-of-focus blur and background noise to achieve reasonable 3D reconstruction with epifluorescence microscopes [12]. In a different fashion, spinning disk confocal microscopy is an alternative to regular confocal microscopy as it overcomes the speed limitations associated with point-scanning by array-scanning the sample. This technique includes a parallel array of illuminated pinholes on a rotating disk, creating a demagnified array of focal volumes. Thus, it is a powerful tool for rapid spatial and temporal imaging in thin and thick specimens [13,14]. Similarly, another approach has been to adapt confocal or two-photon microscopy by coupling a resonant scanner. This galvanometric mirror scanner adapts to the sample’s dynamics, improving spatial and temporal resolution while reducing photobleaching [15].

Light sheet fluorescence microscopy (LSFM) is a technique that provides optical sectioning of the samples by illuminating one slice perpendicular to the detection objective. For example, in single plane illumination microscopy (SPIM) the light sheet is created by passing a laser beam through a cylindrical lens. The advantages of LSFM include good optical sectioning of the whole sample and high temporal resolution, limiting phototoxicity and facilitating live processes imaging [16].

LSFM is a powerful tool for imaging biological processes inside of organoids, including drug uptake [10,17], glucose biosensing [18], cell division dynamic monitoring [16] and spheroid formation [19]. LSFM has also been used to analyze microfluidic devices in applications such as drug screening and precision medicine [20] (for a review, see [21]).

However, standard SPIM configurations have several drawbacks for imaging microfluidic devices. To mitigate these issues, we have designed a microscope that combines features from several previous architectures to optimize SPIM microscopy for its use in microfluidic imaging. First, as the light beam needs to transverse the PDMS walls, significant optical path differences (OPD) can appear due to the different refractive index of the mediums [21,22]. Higher OPD leads to reduced image resolution. To mitigate this issue, we have included an electrically tunable lens (ETL) to compensate for the axial focal shift caused by the optical path difference, as previously described by Xu et al. [23]. Another challenge in standard LSFM is the required movement of the sample. During the scanning phase, the sample is displaced vertically to scan the entire volume [24,25]. However, in microfluidics, this movement can disrupt the equilibrium of the system as the components within the microdevices are in suspension and not fixed inside the microchannels. For this reason, our design eliminates this movement by incorporating a Galvo motor to vertically displace the laser beam coupled with an ETL in the camera turret that adjusts the focal plane to the current location of the light sheet, as previously described [26,27,28].

Besides vertical displacement, rotational movements are commonly used in LSFM to reduce shadowing. Lateral illumination of the samples introduces some shadows when thicker or denser regions of the sample interrupt the light pathway. Rotating the sample can increase the illumination angles and reduce shadowing [10]. These types of movements are acceptable for fixed samples, but in the case of living organisms and, in particular, for microfluidic devices, this movement can lead to additional forces that might affect the precise flow control required for these devices, besides the technical difficulties of connecting pumping devices and outlets to a moving platform. To help reduce the shadowing without moving the sample, we use an additional mirror galvanometer to displace the laser beam parallel to its direction and pivoting cylindrical lens to maintain the angle of the sheet incident on the sample [29].

In this paper, we thus propose an upright SPIM configuration with two Galvo motors that displace the laser plane vertically and laterally to scan the sample and reduce shadowing, respectively. In addition, we also include two ETLs to reduce the OPD effect caused by the PDMS and help with the vertical scanning of the sample. The combination of these system adaptations aims to facilitate the use of LSFM in the field of microfluidics and organ-on-chip technologies. It will allow in situ and in vivo tracking of cell populations and organoids that could be applied in drug screening and disease model characterization [30,31,32].

## 2. Materials and Methods

### 2.1. Imaging System Components and Architecture

**Figure 1:**
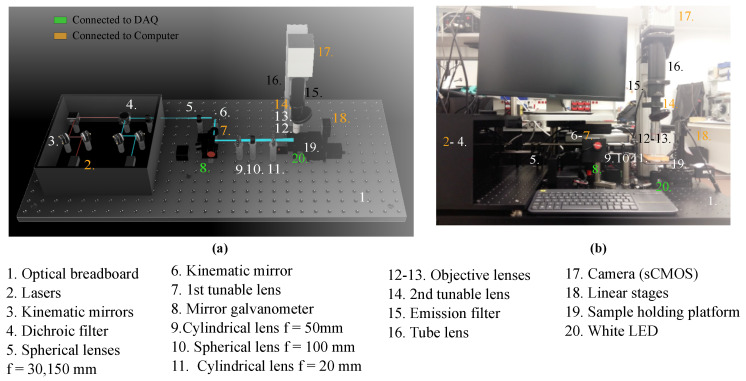
SPIM architecture and components. The model information for all numbered imaging components are listed in Table 1. Those controlled directly by the computer are labeled in orange and the ones controlled by the DAQ appear in green. (**a**) 3D model using Autodesk Fusion 360. CAD files for the components were obtained from the Thorlabs webpage and reproduced with permission. Copyright © 2020 Thorlabs. (**b**) Real system set up at the laboratory. It additionally includes the computer as control center. Components 2–4 are covered to protect the laser system and 6–7 are covered by the Galvo (8).

SPIM microscopy is characterized by the creation of a light sheet after a laser beam crosses a cylindrical lens. A new architecture of the SPIM system has been developed, as shown in Figure 1. The Data Acquisition Device (DAQ) (NI 781443-01 1 2946) and the computer are the control centers of the platform. The individual components of this custom-made SPIM are detailed in Table 1.

#### 2.1.1. General Setup

All the elements of the SPIM platform were mounted on two aluminum optical breadboards of sizes 600 × 900 mm (Thorlabs PBG51506) and 450 × 600 mm (Thorlabs MB4560) to minimize thermal instabilities and provide damping against vibrational effects to optimize the image quality. Posts (Thorlabs RS50/M), rods (Thorlabs ER6) and cage systems (Thorlabs CP02/M) are used throughout the platform as pillars to hold components in the same optical plane onto the breadboard. The sample is placed on a holding platform (ZABER AP102B) directly connected to three linear stages (Zaber T-LSM050A motor), to allow for translation in 3 degrees of freedom and navigation under the objective.

#### 2.1.2. Excitation Path

Two lasers (iBeam smart Toptica Photonics) are the illumination sources of the system, with wavelengths of 488 nm and 635 nm, a diameter of 1 mm, and a maximum power output of 100 mW. Additionally, an LED placed below the sample is available as a supplementary source of white light in the acquisition. The lasers are aligned using two kinematic mirrors (Thorlabs KCB1/M) that adjust the reflection angles and ensure the beam’s collimation in the same optical axis. The next component encountered in the excitation path is a long-pass dichroic filter (Edmund optics), where the 488 nm laser is reflected and the 635 nm laser is transmitted.

Subsequently, Keplerian beam expansion is carried out from 1 to 5 using two achromatic plano-convex spherical lenses with a focal spot of 30 mm (Thorlabs AC254-030-A-ML) and 150 mm (Thorlabs AC254-150-A-ML), respectively. At this point, the optical axis of the enlarged beam is shifted to a 90° angle after it encounters a cage-mounted kinematic mirror (Thorlabs KMSS/M). The next component in the optical plane is the first tunable lens (Edmund EL-16-40-TC-VIS-5D-C), which allows fine changes in the focal spot to ensure the beam is focused under the objective and improve the acquisition focus.

Lastly, the laser crosses the Galvo (Thorlabs GVS012/M), a system of two motorized mirrors that grants 3D scanning of the sample and shadow reduction without moving the sample stage, avoiding unnecessary forces acting on the microfluidics platform. The laser beam outputs at a 90° angle, returning to the initial optical axis orientation.

After exiting the Galvo, three lenses are confronted: A plano-convex cylindrical lens (Thorlabs LJ1695RM-A ) with a focal length of 50 mm that creates the light sheet; a spherical lens with a focal spot of 100 mm (Thorlabs AC254-100-A-ML) to focus the laser under the objective and a second cylindrical lens with a focal length of 20 mm (Thorlabs LJ1328L2-A), that redirects the light sheet onto the focal spot. Finally, the light sheet illuminates a section of the sample laterally, ensuring low phototoxicity compared to confocal systems.

#### 2.1.3. Emission Path

Light is emitted by the sample and recorded by the camera turret, placed perpendicularly to the excitation plane. The camera turret encompasses components that are connected within a tube system. The first element is the objective lens, which in the current setup can be a 5×. Air Plan Apochromat objective (Mitutoyo 378-802-6) or a 20× Air Plan Apochromat Long Working Distance objective (Edmuld Optics 59-878). These can be changed using the objective wheel (Thorlabs OT1), next in the system. Subsequently, a second tunable lens ensures the coordinated change in focus in the z-direction (vertical) as the laser scans the sample, improving the 3D scanning control system. The next component in the turret is a dual band-pass emission optical filter (Edmund optics), with band-passes of 577 ± 12 nm and 690 ± 25 nm, corresponding with the emission wavelengths of the fluorophores in use. Moreover, a tube lens (Thorlabs TL200-CLS2) focuses the image on the camera sensor. A tube lens is a spherical lens with an image distance set to infinity that refocuses the parallel rays that exit the objective lens into a digital sensor, the camera of the system. A digital scientific CMOS (Hamamatsu sCMOS C13440-20CU) was used. The camera in place has an acquisition frequency of up to 100 frames per second (fps), a 2048pixels×2048pixels sensor and a dynamic range of 37,000:1.

#### 2.1.4. Software

The main software that controls the system for image acquisition and all the hardware was provided by Planelight, S.L. The software controls the Galvo motors and the linear stages through the DAQ, as well as directly controlling the voltage sent to the electrically tunable lenses to change their focus positions. The software also includes a first post-processing stitching algorithm to reconstruct the SPIM 3D images. Images were then processed with ImageJ (ImageJ bundled with 64-bit Java 1.8.0).

### 2.2. Sample Preparation

#### 2.2.1. Microfluidic Device Fabrication

Microfluidic chips were fabricated using standard soft lithography techniques. Briefly, the designed microchannels made with AutoCAD were transferred to an SU-8 sheet spin-coated to the desired height by applying UV light through a negative patterned mask. The wafer was used to cast PDMS using soft lithography. PDMS (Sylgard 184) in a 9:1 proportion between elastomer and curing agent was poured on the wafer, degassed in a vacuum chamber, and baked for 2 h at 70 °C to enhance polymerization. After curing and cleaning the surfaces and piercing the inlets and outlets (1.5 mm), PDMS chips were sealed to a glass coverslip using air plasma treatment.

#### 2.2.2. Spheroid Preparation

Spheroids were prepared using fluorescent HaCaT/(H2B-GFP) cells, modified to express green fluorescence in the nucleus. The cells were cultivated in DMEM (Invitrogen Life Technologies) supplemented with 10% fetal bovine serum (Thermo Scientific HyClone) and 1% antibiotic/antimycotic (Thermo Scientific Hyclone) in an incubator at 37 °C and 5% CO_2_ until they reached an 80% confluence. Spheroids were produced following the reference protocol from [33].

#### 2.2.3. Skin Dermal Compartment Preparation

Human fluorescent fibroblasts, transfected with pLZRS-IRES-EGFP vector to express green fluorescence (HF-GFP) in the cytoplasm, were cultured and detached in the same conditions as the HaCaT cells. A skin dermal compartment was prepared using fibrin hydrogels derived from human blood plasma following the protocols described in detail in [34]. Briefly, for a final volume of 500 μL, human blood plasma is added to a final fibrin concentration of 1.2 mg/mL together with antifibrinolytic agent Amchafibrin (Meda Pharma SL) at 0.008% (*w/v*) final concentration. Then, a cell suspension containing HF-GFP was added at a density of 18,000 cells/mL. Immediately after, CaCl_2_ (1%) was added at a final concentration of 0.08% (*w/v*) to trigger fibrin polymerization. NaCl (0.9%) was added to adjust the final volume to 500 μL; the mixture was introduced in a PDMS chip and incubated for 1 h at 37 °C in an atmosphere containing 5% CO_2_ at a 40% relative humidity for hydrogel gelation. Then, the medium was added through a pumping system for 24 h before imaging to allow the fibroblasts to stretch and recover their normal shape. In time-lapse experiments, chips were continuously fed with fresh culture media using a pumping system and maintained at 37 °C and controlled humidity conditions. To promote cell movement within the chip, culture media at two different serum concentrations (0.5 and 20% FBS) were introduced at 5 μL/min in each of the lateral channels using a pumping system.

## 3. Results

### 3.1. Microscope Setup and Characterization

The final configuration of the microscopy system is shown in Figure 1b.

**Figure 2:**
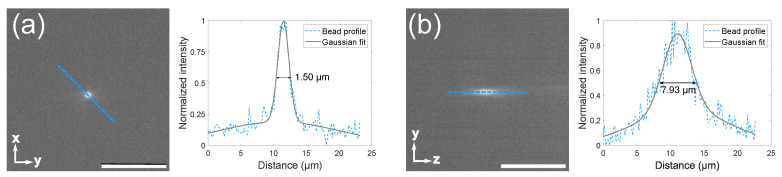
Resolution of the system. The graphs represent the Gaussian function (black) that fits the average microsphere profile together with the original data (blue), obtained from the diagonals passing through the highest intensity pixel: (**a**) x-y cross-section of a 1 µm diameter microsphere; (**b**) x-z cross-section of a 1 µm diameter microsphere, both imaged with a 5× Air Plan Apochromat objective.

Two different objectives were characterized, a 20× and a 5×, leading to a field of view of 0.39 mm × 0.39 mm and 1.61 mm × 1.61 mm, respectively. The full width at half maximum (FWHM) of the point spread function (PSF) of a fluorescent microsphere was used to characterize the spatial resolution of the system. Microspheres of 0.5 μm and 1 μm diameter were diluted to 1:1000 of the stock concentration in distilled water and imaged using the 488 nm laser. From the cross-sectional intensity profiles of the imaged spheres, the final PSF, FWHM, and spatial resolution was obtained, with an example seen in Figure 2.

The resulting spatial resolution was 2.46±0.60 μm in the x and y axes and 9.28±2.05 μm in the z-direction using the 5× objective and 1 μm microspheres. Using the 20× objective to image 0.5 μm microspheres gave a resolution of 1.50±0.43 μm in the x and y axes and of 7.93±1.41 μm in the z-direction.

### 3.2. Imaging of Spheroids

**Figure 3:**
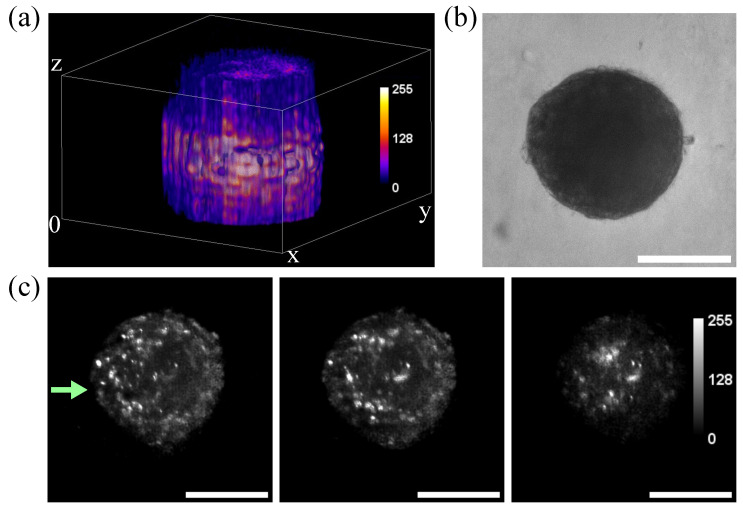
Volumetric reconstruction of spheroids prepared with fluorescent HaCaT/(H2B- GFP) cells and acquired with the custom-made SPIM microscope: (**a**) Reconstruction of spheroid generated using the Fiji plugin Volume Viewer, with volume rendering using trilinear interpolation and Fire look-up table (LUT); (**b**) Bright-field microscopy image of the sample, acquired using the LED in the SPIM; (**c**) Axial cross-sectional cuts of the spheroid at different heights along the z axis. The green arrow represents the direction the light is coming from. Scale bars: 100 μm.

One of the main applications of LSFM microscopy is the fast acquisition of three-dimensional volumes [35,36]. The capability of the designed setup to image macroscopic biological samples was demonstrated using non-fixed spheroids, prepared with fluorescent HaCaT/(H2B- GFP) cells, and imaged using the 488 nm laser line in the SPIM system, as observed in Figure 3. The system permits us to distinguish the nuclei of the different cells conforming to the spheroid in cross-sections located at different heights within the spheroid (Figure 3c). This leads to the possibility of performing 3D reconstructions of living samples. Some reduction in intensity is expected in the areas further away from the light source (represented in Figure 3c by the green arrow) due to the relative opacity of the spheroid itself, which can also be appreciated in Figure 3b. Some intensity in those distant areas was recovered using the light sheet rotation provided by the Galvo motors, as can be appreciated in Figure 3c where individual nuclei can be distinguished in the further regions. This represents a substantial advantage as it does not require tissue fixing and histological processing for whole sample characterization and imaging.

### 3.3. Imaging of Fibroblasts-on-a-Chip

**Figure 4:**
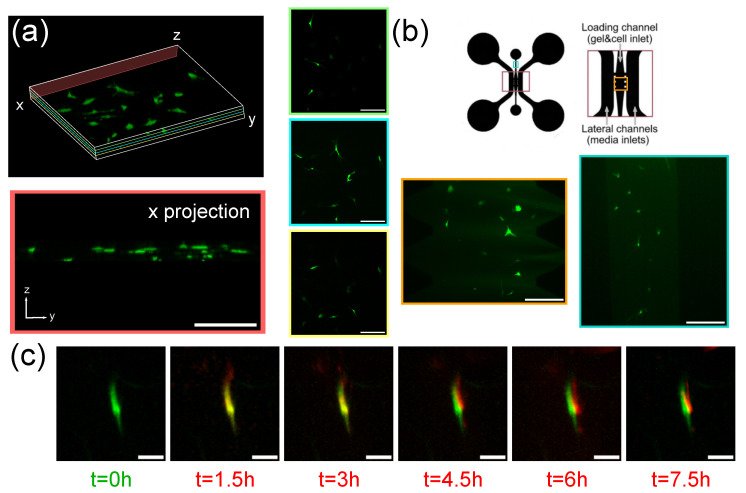
Skin dermal fibroblasts imaged growing in a plasma gel. (**a**) Volumetric reconstruction of fibroblasts embedded in a hydrogel acquired with the custom-made SPIM microscope. Three cross-sections in the xy plane are shown (green, blue and yellow) at different heights, as well as a projection in the x direction to show the relative z positions of the cells (red). Scale bars: 100 μm; (**b**) Scheme of the microfluidic device where the fibroblasts are embedded with channels outlined. Fibroblasts are shown growing in the middle channel both at the entrance (blue) as well as in the middle region (orange). Scale bars: 200 μm; (**c**) Time lapse of a fibroblast surveying its environment inside of the microfluidic channel with the initial position shown in green and successive positions shown in red. Scale bars: 20 μm. All reconstructions were generated using the Fiji plugin Volume Viewer, with volume rendering using trilinear interpolation.

The functionality of the SPIM setup, which was tested for both 3D volume reconstructions and long-term sample imaging, was also analyzed using in vitro dermis models. We show that the system can image fibroblast at distinct heights within a plasma-derived fibrin hydrogel (Figure 4a), as well as fibroblasts growing in the same fibrin gel placed inside a microfluidic device (Figure 4b, device reproduced from previous research by Anguiano et al. [37]). The device includes one central channel where the gel is formed as well as two lateral channels for cell feeding. In this case, the height of the channel was in the range of the size of the fibroblasts [38], so there were no changes in height between the different cells (Figure 4b). To test the feasibility of this experimental setting for lifetime imaging and cell tracking, culture media with high (20%) and low (0.5%) serum concentrations were introduced in each of the parallel channels to form a nutrient gradient and induce cell migration towards the higher nutrient regions. Cell movement in the XY-plane towards the channel containing high serum concentration was proven in the first 7 h (Figure 4c).

### 3.4. Imaging of Fluorescent Microbeads in Zero-Flow Chambers

**Figure 5:**
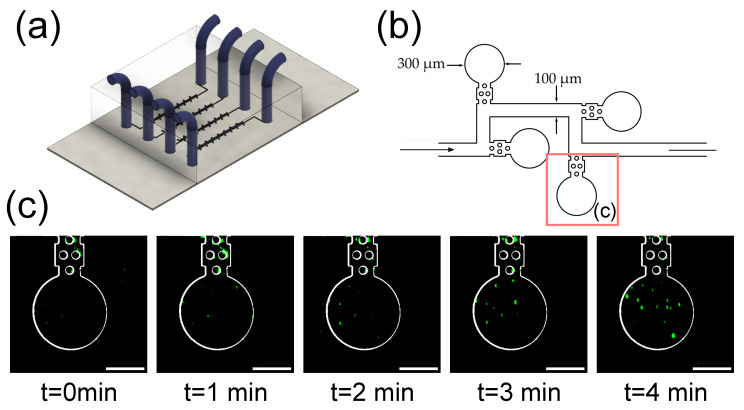
Microbeads being entrapped in microfluidic chambers. (**a**) Scheme of the microfluidic device used for zero-flow bacterial entrapping; (**b**) Detail of the microfluidic channel showing the zero-flow chambers where bacteria are loaded (left side), and subsequently, media can be passed from the feeding direction (right side) and reached the entrapped bacteria by diffusion; (**c**) 1 μm fluorescent bead being trapped inside the microfluidic chamber. The microfluidic device outlines have been obtained from the brightfield image. Scale bar: 100 μm.

Finally, this last section focuses on the imaging of fluorescent microbeads filling zero-flow microfluidic chambers (Figure 5a,b). The objective of this device (based on the design published by Sun et al. [39]) is to trap bacteria in the microchambers and use it as a multiplexed antibiotic testing platform, where each parallel channel contains a different combination of bacterial strain and potential drug compound. The bacteria are introduced from one end, and then the culture media with the tested compounds are introduced from the other end, ensuring zero flow in the cambers, that receive the nutrients and compounds by diffusion. To demonstrate the ability of the system to image these types of devices, fluorescent microbeads were introduced in the channels in the filling direction, simulating the filling of the chambers by bacterial cells. A time lapse image of a chamber filling with fluorescent microbeads is shown in Figure 5c. It is worth noting that although the curved walls of the chambers are in the path of the light sheet, the optical density of the PDMS and the culture media is similar enough that there is no relevant light scattering at the interface.

## 4. Discussion

A fast upright SPIM microscopy architecture able to acquire fluorescent 3D images of live biological samples has been described, as well as several applications for its use with microfluidic devices. Its components and setup have been detailed, including two Galvo motors to scan the sample and two tunable lenses to optimize sample focusing. These are key to understanding the improvements of this architecture with respect to the state of the art. Recent manuscripts show how SPIM can acquire high-resolution images with low-phototoxicity [40,41]. In particular, the inclusion of the two Galvo motors allows for imaging with a static sample stage. A first Galvo motor that scans the sample in the vertical direction, coupled with an ETL in the detection turret allows the laser plane to scan the entire sample (up to 28 mm) without vertically displacing it. On a similar note, previous work shows that sample rotation is required for 3D imaging free of shadows from opaque regions of the sample [10]. However, the need to rotate the sample limits its applications in live imaging of suspended or in-motion samples. The solution we propose overcomes this limitation by controlling the lateral incident angle of the light sheet that mitigates the effects of shadowing. Although this method only provides a limited range of illumination angles, in practice, we see a reduction in shadowing without the technical complications of sample rotation and image reconstruction required for previous designs. Currently, all of the electronic components are controlled by proprietary software. However, the main contribution of this work is in the optical hardware, and could be implemented with any of the open software developed for SPIM control in the past decade [42,43,44].

Regarding image resolution, the system achieves a micrometer-level resolution of 1.50±0.43 μm in the xy plane and of 7.93±1.41 μm in the z-direction alongside a field of view of 0.39 mm × 0.39 mm for the 20× objective, granting its applicability in biological studies. Higher magnification objectives could be mounted on the objective wheel if higher resolution in the xy plane was required. The achieved resolution in the plane perpendicular to the laser beam is limited by the width of the light sheet used for imaging, and in the current configuration can be used for cell-level imaging. To image subcellular details, a thinner laser plane can be obtained by adjusting the system of lenses used in beam expansion. Expanding the beam diameter will in turn reduce the laser sheet width, improving the z-resolution. The user should be aware that our setup requires the sample holder to be transparent and to have a flat lateral surface so that the laser plane falls perpendicularly onto the surface and undesirable scattering is avoided. Generally, it should be possible to find such a holder for standard biological samples. This issue can be overcome for PDMS microfluidic devices by preparing the PDMS devices using a mold with regular walls instead of using a manual cutter. For microfluidic designs that include walls in the light path that are not perpendicular to the laser plane, some scattering is expected, as the refractive index of PDMS is 1.43 and the one of standard culture media is 1.34 [45]. However, as shown in Figure 5c, this effect does not significantly affect image acquisition. Nevertheless, refractive index matching culture solutions could be used to reduce the diffraction coming from the interface between the PDMS and the culture channels [23].

Historically, most in vitro experiments were carried out on 2D cellular cultures, which present substantial limitations in their inability to reproduce the in vivo physiology of the tissues. Three-dimensional cultures overcome some of these constraints by providing information about morphology, connectivity, tissue architecture and physiology [46]. Some examples of these constructs include hydrogel cultures, organs-on-chips, organoids, and spheroids. Among the different types of 3D cell culture systems, spheroids are spherical aggregates of cells relevant to the study of tumor models and stem cell research [47]. In oncology studies, spheroids present a much more realistic representation of the in vivo tumors than traditional 2D cell cultures, which makes them a useful tool for assessing novel cancer therapies [48].

In the case of organs-on-a-chip, the goal is to reproduce the smallest functional unit of a tissue or organ. In our device, the hydrogel models the dermis three-dimensional environment in which fibroblasts grow within the body. Imaging shows fibroblasts growing in a three-dimensional network within the channel, with different cells located at different layers within the gel. The static sample holder used in this microscope configuration is useful to image living spheroids that are growing embedded in media and could potentially move during the process. However, it is critical for organs on a chip, as it solves two main issues. On one hand, it is impractical to have the chips move while connected to an external pump that is providing flow within the channels. Additionally, the movement of the chip would produce external forces that could affect the flow inside the channels, which is highly controlled to produce the desired objectives.

The high-speed camera used, with an imaging rate of 100 fps, can be used to image fast cellular processes, as well as reduce the acquisition time, which allows live imaging of cells without an environmental chamber. A custom-made environmental chamber was used for longer imaging periods in this work, but the setup has enough room to include a standard environmental chamber if the applications would require it.

## 5. Conclusions

The novel LSFM architecture proposed allows the combination of SPIM microscopy techniques with microfluidic devices, particularly for cell culture purposes, as shown by the imaging of two different 3D living samples and the acquisition of 2D images for bead trapping in zero-flow chambers. In the 3D acquisitions, no rotation of the sample was required, which significantly eases the manipulation of liquid samples such as the spheroids. The imaging of different planes allows visualization of volumes of living samples, particularly, HaCaT spheroids and a dermis-like structure of fibroblasts embedded in a plasma-derived fibrin hydrogel.

Thus, the compatibility of the set-up with the use of microfluidic devices for biomedical research is demonstrated, as well as its resolution and image quality with no observable scattering or shadows.

## Figures and Tables

**Table 1:** Components and models of the SPIM system in Figure 1.

Number	Component	Model
1	Optical breadboard	Thorlabs PBG51506
2	Lasers	iBeam smart Ultra Compact
3	Kinematic mirrors	Thorlabs KCB1/M
4	Dichroic filter	Edmund optics 25mm Diam.
5	Spherical lenses,	Thorlabs AC254-030-A-ML
	f=30,150mm	Thorlabs AC254-150-A-ML
6	Kinematic mirror	Thorlabs KMSS/M
7	1st tunable lens	Ed. EL-16-40-TC-VIS-5D-C
8	Mirror galvanometer	Thorlabs GVS012/M
9	Cylindrical lens, f=50mm	Thorlabs LJ1695RM-A
10	Spherical lens, f = 100 mm	Thorlabs AC254-100-A-ML
11	Cylindrical lens, f = 20 mm	Thorlabs LJ1328L2-A
12	Objective lens 5×	Mitutoyo 378-802-6
13	Objective lens 20×	Edmund Optics 59-878
14	2nd tunable lens	Ed. EL-16-40-TC-VIS-5D-C
15	Emission filter	Edmund optics Dual-Band
16	Tube lens	Thorlabs TL200-CLS2
17	Camera (sCMOS)	Hamamatsu C13440-20CU
18	Linear stages	Zaber T-LSM050A
19	Sample holding platform	ZABER AP102B
20	White LED	

## Data Availability

The data presented in this study is available within the published article.

## Abbreviations

The following abbreviations are used in this manuscript:

LSFM	Light sheet fluorescence microscopy
SPIM	Single-plane illumination microscopy
PDMS	Polydimethylsiloxane
OPD	Optical path differences
ETL	Electrically tunable lens
DAQ	Data acquisition device
fps	frames per second
FWHM	Full width at half maximum
PSF	Point spread function
LUT	Look up table
